# Rapid identification of haloarchaea and methanoarchaea using the matrix assisted laser desorption/ionization time-of-flight mass spectrometry

**DOI:** 10.1038/srep16326

**Published:** 2015-11-06

**Authors:** Chao-Jen Shih, Sheng-Chung Chen, Chieh-Yin Weng, Mei-Chin Lai, Yu-Liang Yang

**Affiliations:** 1 Agricultural Biotechnology Research Center, Academia Sinica, Taipei, Taiwan; 2 Department of Life Sciences, National Chung Hsing University, Taichung, Taiwan;; 3 Agricultural Biotechnology Center, National Chung Hsing University, Taichung, Taiwan; 4 Ph.D. Program in Microbial Genomics, National Chung Hsing University and Academia Sinica, Taiwan

## Abstract

The aim of this study was to classify certain environmental haloarchaea and methanoarchaea using matrix assisted laser desorption/ionization time-of-flight mass spectrometry (MALDI-TOF MS), and to expand the archaeal mass spectral database. A total of 69 archaea were collected including type strains and samples isolated locally from different environments. For extraction of the haloarchaeal total cell peptides/proteins, a simple method of acetonitrile extraction was developed. Cluster analysis conducted with the MALDI-TOF MS data overcame the high divergence in intragenomic 16S rRNA sequences in haloarchaea and clearly distinguished *Methanohalophilus mahii* from *M. portucalensis*. Putative biomarkers that can distinguish several particular archaeal genera were also assigned. In conclusion, this study expands the mass spectral database of peptide/protein fingerprints from bacteria and fungi to the archaea domain and provides a rapid identification platform for environmental archaeal samples.

The Archaea represent the third domain of life, and are distinguished from bacteria by the 16S ribosomal RNA sequences^1^,^2^. They share similar morphology to bacteria but possess several genetic and metabolic characteristics closely related to eukaryotes, such as their transcription and translation mechanisms. Archaea were initially considered to be extremophiles that live in various harsh environments, such as hot springs and salt lakes. However, they have now been demonstrated to exist in various habitats including marshlands, sewages, oceans, and soils as well as the intestinal tract of animals^3^. Most of the cultured and widely-studied archaea species belong to the phylum of Euryarchaeota which contains methanoarchaea and haloarchaea. Multiple copies of rRNA operons are often found in haloarchaea^4^,^5^. For instance, *Haloarcula marismortui* contains three rRNA operons, rrnA, rrnB, and rrnC. The 16S rRNA genes of operons B and C share 99.3% sequence identities. However, the 16S rRNA gene from operon A has a highly divergent nucleotide sequence; 94.8% identity with operon B, and 94.4% identity with operon C^6^. This high divergence in the intragenomic 16S rRNA sequences in haloarchaea makes it difficult to quickly identify newly isolated haloarchaea species based on 16S rRNA sequences.

Matrix assisted laser desorption/ionization time-of-flight mass spectrometry (MALDI-TOF MS) is an emerging technology in clinical microbiology^7^. This relatively low-cost and quick method is currently widely used for the rapid identification of pathogenic microorganisms, including bacteria^8^,^9^,^10^,^11^,^12^,^13^, fungi^11^,^14^,^15^,^16^,^17^, and even viruses^18^, in clinical microbial laboratories. However, only a few reports have applied this method to identify environmental microbes, such as archaea^19^,^20^. Krader and Emerson^20^ used MALDI-TOF MS to identify 28 archaea (four methanoarchaea genera and three haloarchaea genera) and some extremophilic bacteria by detecting the cell wall components ranging from 500 to 3000 Da. However, the haloarchaea and most methanoarchaea do not have murein-based cell walls and the mass range detected in their study is not adopted by the current method for microbial identification, because many secondary metabolites also fall in this mass range. Recently, a total of 13 archaea strains including four human-associated methanoarchaea, *Methanobrevibacter smithii*, *Methanobrevibacter oralis* and *Methanosphaera stadtmanae* as well as *Methanomassiliicoccus luminyensis*, have been identified using the MALDI-TOF MS^19^, demonstrating that MALDI-TOF MS is first-line technique capable of identifying human methanoarchaea.

The overall goal of this study was to evaluate the application of MALDI-TOF MS for the identification of haloarchaea and methanoarchaea. We established a method and database for rapid identification of the newly isolated archaea, and overcame the problems of intragenomic 16S rRNA sequence divergence that have hitherto complicated haloarchaea identification. Moreover, the specific signals in the MALDI-TOF MS fingerprints which could be used to differentiate the tested archaea were assigned as putative molecular biomarkers of archaea to achieve more efficient classification using this methodology.

## Results

### Sample preparation

**Figure 1:**
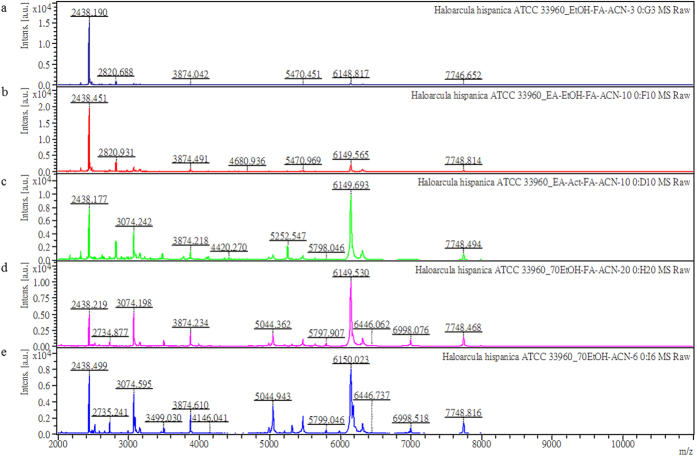
Comparison of the haloarchaea, *Haloarcula hispanica* ATCC 33960^T^ mass spectra from samples that used different extraction methods. (**a**) The cell pellet was suspended in sterile water and then ethanol was added to precipitate the peptides/proteins. The pellet was suspended in 70% FA then ACN was added to give a final concentration of 50%. (**b**) The cell pellet was suspended in sterile water then EA was added and mixed thoroughly. The EA layer was discarded and ethanol was added to the water layer to precipitate the peptides/proteins. The pellet was suspended in 70% FA then ACN was added to give a final concentration of 50%. (**c**) A similar procedure as outlined in b was used, but ethanol was substituted by acetone to precipitate the peptides/proteins. (**d**) The cell pellet was directly suspended in 75% ethanol followed by the same procedure as outlined in a (**e**) The cell pellet was directly suspended 75% ethanol then the precipitated pellet was extracted by ACN.

In this study, several type strains of haloarchaea, halophilic methanoarchaea, halotolerant methanoarchaea and non-halophilic methanoarchaea, together with some locally isolated environmental methanoarchaea and haloarchaea were collected for MALDI-TOF MS and 16S rRNA analysis^21^,^22^,^23^,^24^,^25^,^26^,^27^,^28^. Because of the extremely high intracellular osmotic pressure, the haloarchaea cells were easily lysed by water during the traditional peptide/protein extraction process. The released viscous nucleic acids and long chain fatty acids made it difficult to extract the peptide/proteins from the sticky solution for MALDI-TOF MS analysis (Fig. 1a). Some modified extraction methods were applied to *Haloarcula hispanica*, for example, the long chain fatty acid was removed using ethyl acetate (EtOAc) before the peptide/protein precipitation using 70% ethanol (Fig. 1b). Peptide/protein precipitation was conducted by using acetone instead of 70% ethanol and more peptide/protein signals were observed (Fig. 1c). We then simplified the procedure to extract the peptides/proteins by directly suspending cell pellets using 75% ethanol followed by 70% formic acid and finally 50% acetonitrile (ACN) (Fig. 1d). The simplest procedure was to suspend the cell pellets and use ACN alone to extract the peptides/proteins (Fig. 1e). Using this method, the MALDI-TOF MS fingerprints observed were almost identical to those observed by using EtOAc extraction followed by acetone precipitation (Fig. 1c).

### MALDI-TOF MS analysis of the haloarchaea

**Figure 2:**
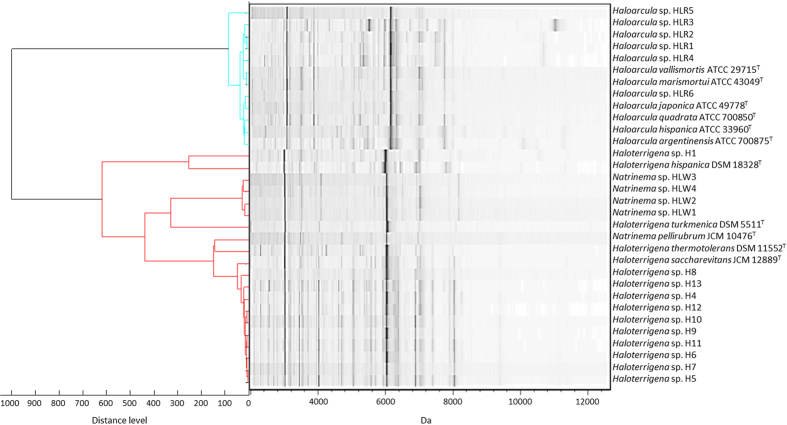
MSP dendrogram and spectra gel view of the haloarchaea including type strains and the local isolated strains created by the protein mass spectra.

A total of 32 haloarchaea were collected including type strains and samples isolated locally from the salterns in Taiwan (Table 1). The main spectra library (MSP) dendrogram was deduced from the MALDI-TOF MS fingerprints of the haloarchaea using the EtOAc-acetone extraction method (Fig. 1c). The haloarchaea were differentiated into two groups: the genus *Haloarcula* and the genera *Haloterrigena*/*Natrinema* (Fig. 2). The distributions of the haloarchaea type strains in the MSP dendrogram and the 16S rRNA phylogenetic tree were almost identical (Fig. 2 and Supplementary Fig. S1).

**Figure 3:**
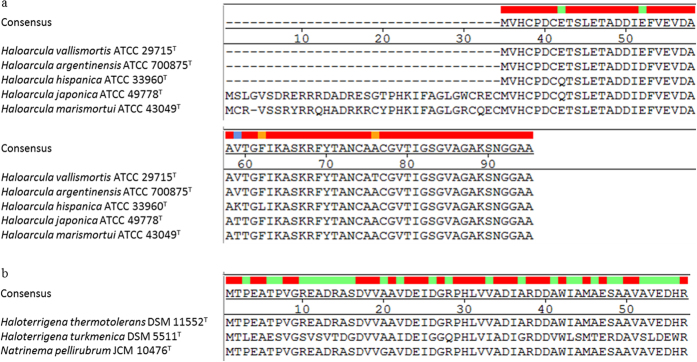
Sequence alignment of the putative biomarkers. (**a**) *Haloarcula* species and (**b**) *Haloterrigena* species and *Natrinema pellirubrum*.

Candidate molecular biomarker assignment is an important aspect of mass spectrometric-based identification techniques and has been successfully applied to different bacterial species^29^,^30^,^31^,^32^. The significant signals, around 6.0–6.1 kDa in the MALDI-TOF MS fingerprints of haloarchaea were observed and used to differentiate haloarchaea into two obvious groups (Fig. 2). The assignments of these mass signals of the haloarchaea type strains are presented in Table 2. All of the putative targets were uncharacterized proteins or hypothetical proteins, for example, M0KI28, G0HR47, EMA34946, YP_137385, and M0JI28 that belonged to the genus *Haloarcula* and shared high amino acid sequence identity. L0JI54, YP_003403049 as well as M0BND2 shared high sequence identities and belonged to the genera *Natrinema* and *Haloterrigena*. The sequence alignment of these putative target signals is presented in Fig. 3.

### MALDI-TOF MS analysis of the methanoarchaea

**Figure 4:**
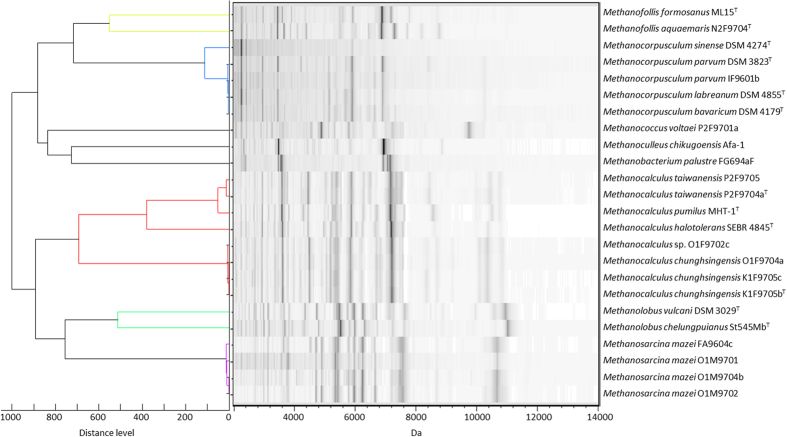
MSP dendrogram and spectra gel view of the halotolerant and non-halophilic methanoarchaea including type strains and the locally isolated strains created by the protein mass spectra.

**Figure 5:**
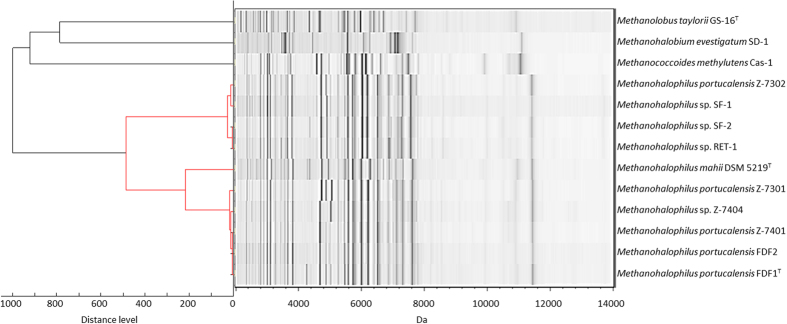
MSP dendrogram and spectra gel view of the halophilic methanoarchaea including type strains and the locally isolated strains created by the protein mass spectra.

Twenty-four halotolerant and non-halophilic methanoarchaea were collected including type strains and locally isolated samples from different environments in Taiwan. The MSP dendrogram deduced from the MALDI-TOF MS fingerprints successfully distinguished the methanoarchaea as different groups with different genera. The clustering results of MSP dendrogram were almost identical to those of the 16S rRNA phylogenetic analysis (Fig. 4 and Supplementary Fig. S2). In addition, 13 halophilic methanoarchaea were surveyed in this study (Table 1) and five of them belonged to the same species, *Methanohalophilus portucalensis*, based on the phenetic characters, DNA reassociation and denaturing electrophoresis of whole-cell proteins^33^. Interestingly, these five strains were clustered into two groups in the MSP dendrogram analysis (Fig. 5), which also matched the results in 16S rRNA phylogenetic analysis (Supplementary Fig. S3).

Among the methanoarchaea selected in this study, only three sets of whole genome sequence data, *Methanosarcina mazei* Gö1^T^ (NC_003901)^34^, *Methanococcus voltaei* A3 (NC_014222, direct submission), and *Methanohalophilus mahii* DSM 5219^T^ (NC_014002, direct submission), are available for the identification of specific signals in MALDI-TOF MS fingerprints. The specific signals of the *Methanosarcina mazei* strains (10.6 kDa) and *Methanococcus voltaei* P2F9701a (9.7 kDa) were tentatively assigned as 50S ribosomal proteins L31e and L12 (Fig. 4 and Table 2). The other genus- or species-specific signals, such as 6.9 kDa of genus *Methanofollis*, 5.9 and 6.9 kDa of genus *Methanocorpusculum*, 6.9 kDa of *Methanoculleus chikugoensis* Afa-1, 6.9–7.2 kDa of *Methanobacterium palustre* FG694aF, 7.2 kDa of genus *Methanocalculus*, 10.9 kDa of *Methanolobus vulcani* DSM 3029^T^, 11.0 kDa of *M. chelungpuianus* St545Mb^T^, 11.4 kDa of genus *Methanohalophilus*, 11.1 kDa of *Methanococcoides methylutens* Cas-1, and 6.9–7.2 kDa of *Methanohalobium evestigatum* SD-1 were observed and shown in Figs 4 and 5.

## Discussion

The aim of this study was to evaluate the application of MALDI-TOF MS to haloarchaea and methanoarchaea identification and to establish the database for identification of newly isolated archaea strains. Dridi^19^ *et al.* demonstrated that the MALDI-TOF MS fingerprints ranging from 3–20 kDa were capable of classifying haloarchaea, thermophilic archaea, and methanoarchaea. The number and diversity of archaea strains for MALDI-TOF MS evaluation was extended in this study. Peptide/protein extraction methods have been demonstrated to have a significant impact on the quality of MALDI-TOF MS fingerprints^10^,^35^,^36^. Here we revealed that interference of long chain lipids and cell lysis due to osmotic pressure seriously compromises the quality of the mass data (Fig. 1). Fortunately, simple extraction using ACN for haloarchaea samples generates high quality MALDI-TOF MS fingerprints for identification.

Haloarchaea often contain more than one copy of the 16S rRNA gene, such as the rrnA and rrnB of *Haloarcula quadrata* ATCC 700850^T^, *Haloarcula* sp. HLR5 and *Haloterrigena* sp. H13 as well as the rrnA, rrnB, and rrnC of *Haloarcula marismortui* ATCC 43049^T^. This high intragenomic 16S rRNA divergence causes difficulty in clearly classifying and identifying newly isolated strains. In this study, identification using MALDI-TOF MS provides a simple and efficient method to overcome the problem of multiple 16S rRNA gene copies.

According to the MSP dendrogram analysis of the haloarchaea, several *Haloterrigena* strains were mixed with *Natrinema* strains (Fig. 2). It has been reported that the genera *Haloterrigena* and *Natrinema* overlap to a large extent based on the 16S rRNA phylogenetic tree and DNA-DNA hybridization^37^,^38^. In this study, the classification of the haloarchaea based on the MALDI-TOF MS fingerprint data also showed that species of *Haloterrigena* and *Natrinema* clustered together and failed to differentiate these two genera (Fig. 2). The 16S rRNA phylogenetic analysis (Supplementary Fig. S1) indicated that *Haloterrigena thermotolerans* DSM 11552^T^ and *Natrinema pellirubrum* JCM 10476^T^ are very likely the same species with 16S rRNA gene sequences similarity at 99.46%. Additionally, genome relatedness between *H. thermotolerans* DSM 11552^T^ (ID16354) and *N. pellirubrum* JCM 10476^T^ (ID11383) was computed using the Average Nucleotide Identity (ANI) with values of 95.6%. An ANI threshold range (95–96%) for species demarcation had previously been suggested and Kim^39^ *et al.* also showed an apparent distinction in the overall ANI distribution between intra- and interspecies relationships at around 95–96% ANI. Results indicated here strongly suggest the requirement of re-nomenclature of genus *Haloterrigena* and *Natrinema*.

Several environmental methanoarchaea including type strains and local isolates were surveyed in this study and were classified well based on their MALDI-TOF MS fingerprints. According to the 16S rRNA phylogenetic analysis, *Methanohalophilus mahii* DSM 5219^T^ shared 99.6% identity with *M. portucalensis* FDF1^T^ (Supplementary Table S1). However, the *in silico* whole genome hybridization (GGDA) result indicated that *Methanohalophilus mahii* DSM 5219^T^ shared lower than 70% identity with *M. portucalensis* FDF1^T^ and this demonstrated that they are different species (M.-C. Lai, unpublished data). The MALDI-TOF MS fingerprints clustered ten selected *Methanohalophilus* strains, including three species, into four major groups (Fig. 5) and the *M. mahii* DSM 5219^T^ was independently isolated from of *M. portucalensis*. In addition, eight *M. portucalensis* strains were successfully differentiated into two clades, which mirrored the 16S rRNA phylogenetic analysis. This demonstrates that MALDI-TOF MS is capable of classifying not only genera and species, but also strains of the same species.

The MALDI-TOF MS fingerprint range from 3–20 kDa is thought to be relatively consistent for microbial identification, since the molecular weights of many ribosomal peptides/proteins are in this region. However, the culture conditions of microbes could affect the mass profiling. Therefore, combining the MALDI-TOF MS fingerprint and molecular biomarkers would provide more information for microbial identification. Candidate molecular biomarkers for mass spectrometric-based identification techniques have been successfully applied to several bacterial species; however, not to archaea. In this study, the whole genome data of several haloarchaea type strains are available for the identification of specific signals in MALDI-TOF MS fingerprints. The original amino acid sequences of the putative biomarkers EMA34946 and YP_137385 were 95 and 94 aa, respectively. We observed that the amino acid sequences at 35–95 aa of EMA34946 and 35–95 aa of YP_137385 shared high identities with those of another three biomarkers, M0KI28, G0HR47 and Q5V7R0 (Fig. 3). The predicted molecular weights of these two partial peptide sequences were very close to the observed mass in MALDI-TOF MS, thus, we proposed that the actual length of polypeptides released from both EMA34946 and YP_137385 were 61 amino acids (Table 2). In addition, the predicted mass of the putative biomarker YP_003403049 shared a 0.93% error with the observed mass, implying that some post translational modifications occur in this protein.

In conclusion, we have developed a simplified sample preparation method for haloarchaea that overcomes the problem of extremely high intracellular osmotic pressure. The MALDI-TOF MS datasets of 69 archaea are provided in the Supplementary Datasets. According to the classifications of the haloarchaea and methanoarchaea, the MALDI-TOF MS fingerprints were able to differentiate genera, species, and even strains of most archaea and the results are comparable to 16S rRNA phylogenetic analysis. We have expanded the MALDI-TOF MS database from bacteria and fungi to the archaea domain and provide a rapid identification platform for environmental archaeal samples. This is the first report tackling the identification of candidate molecular biomarkers for the haloarchaea and methanoarchaea, and provides additional information for the identification of particular archaea genera.

## Methods

### Archaea strains and culture conditions

The archaea strains used in this study were 32 haloarchaea, 13 halophilic methanoarchaea and 24 halotolerant and non-halophilic methanoarchaea (Table 1). The haloarchaea were cultivated in NHA medium, which contained (per liter): 240.0 g NaCl, 10.0 g MgSO_4_·7H_2_O, 5.0 g KCl, 3.0 g trisodium citrate, 1.0 g NaNO_3_, 0.2 g CaCl_2_·6H_2_O, 5.0 g casamino acid, pH 7.2, and incubated at 45 °C with agitation (80 rpm in a horizontal shaker) until the stationary phase^40^. The halophilic methanoarchaea were routinely incubated at 37 °C in defined medium that contained 12% or 4% NaCl and 40 mM trimethylamine as the methanogenesis substrate^41^. The non-halophilic methanoarchaea were routinely incubated at 37 °C in MB/W medium^27^ with different methanogenesis substrates listed in (Table 1).

### Peptides/protein extraction for MALDI-TOF MS

The extraction of halophilic, halotolerant and non-halophilic methanoarchaea peptides/proteins was according to the manufacturer’s recommendations. One milliliter of methanoarchaea culture was transferred into a sterile Eppendorf tube and centrifuged at 17000 × g for 2 min and the cell pellet was suspended in 300 μL of sterile water; 900 μL of absolute ethanol was added and mixed thoroughly. The final concentration of ethanol was 75%. The sample was further centrifuged at 17000 × g for 2 min and the supernatant was discarded. The sample was centrifuged again to completely remove residual ethanol by carefully pipetting. After the pellets were dried at room temperature for several minutes, 50 μL of 70% FA was added and mixed well by pipetting and/or by vortexing then 50 μL of pure ACN was added and mixed carefully. The final concentration of ACN was 50%. After centrifuging at 17000 × g, 1 μL of supernatant including the entire extract was deposited on a TP 384 target plate (Bruker Daltonics, Leipzig, Germany). After the samples were air-dried, 1 μL of matrix solution [saturated solution of α-cyano-4-hydroxycinnamic acid (α-HCCA) in 50% ACN, 2.5% trifluoracetic acid] was covered on the samples and then air-dried for 5 min.

For extraction of peptides/proteins from the haloarchaea, the cell pellet was suspended in 300 μL of sterile water then 300 μL of EA was added and mixed thoroughly to remove the long chain ether linked fatty acids. The EA layer was discarded and 900 μL of acetone to precipitate peptides/proteins was added to the water layer. After centrifuging at 17000 × g for 2 min, the supernatant was discarded and the pellet was suspended in 50 μL of 70% formic acid. The subsequent procedures were the same as described above. A simplified procedure was also used to extract the peptides/proteins by directly suspending cell pellets in 75% ethanol followed by 70% FA and finally 50% ACN. The simplest process, i.e., suspending the cell pellets with 75% ethanol and then extracting by ACN was also tested in this study.

### Archaea MALDI-TOF-MS protein profile database

Twenty four deposits were made, within one TP 384 target plate, for each archaea strain, and peptide/protein profiles were determined with Bruker Autoflex Speed MALDI-TOF/TOF MS (Bruker Daltonics) using FlexControl software. The AutoXecute acquisition control was applied for the automated data acquisition. For each spectrum, 1200 laser shots in 200-shot steps from different positions of the sample spot, were accumulated and analyzed. The parameter of laser size was set as large and the frequency was 1000 Hz. Spectra were collected in the linear positive mode (Detector Gain 13.9 × 2901V) with mass-to-charge ratio (m/z) from 2,000 to 20,000 and processed with parameters including smoothing (Savitzky Golay; width: 5 m/z; cycles: 1), baseline correction (Top-hat), and peak detection (Centroid; signal to noise threshold: 3; maximal number of peaks: 100; peak width: 5 m/z) using Biotyper 3.1 software and library (version 3.1.66, with 4,613 entries; Bruker Daltonics). The peptides/proteins extract of *Escherichia coli* DH5α was used as a positive control and the identified scores against Biotyper 3.1 library must be higher than 2.300. The matrix-only well was a negative control.

Ten to twenty four replicate spectra of each archaea strain, with proportion of reproducible peaks higher than 0.6, were selected to calculate a reference spectrum using the automated major spectra projection (MSP) function of Biotyper 3.1 software. For re-identification of each subculturing strain, three replicate spectra were used to against the reference spectrum. The re-identified scores and variations of all strains were shown in Table 1. Re-identification, clustering analysis and generation of a MSP dendrogram of the archaea strains were also conducted using Biotyper 3.1 software.

The distance level of the MSP dendrogram was calculated from three separate values for three fundamental characteristics of the sample and the reference spectra. First, the numbers of signals in the reference spectrum that had a closely matching partner in the candidate spectrum were calculated. No matches returns a value =0 and a complete match returns a value =1. Then, the numbers of signals in the candidate spectrum that have a closely matching partner in the reference spectrum were calculated. No matches returns a value =0 and a complete match returns a value =1. Finally, the symmetry of the matching signal pairs is computed. If the high-intensity signals of the candidate spectrum correspond with the high-intensity signals of the reference spectrum and the low-intensity signals also correspond, this result in high symmetry value and the so-called correlation matrix yields a value close to 1. If the matching pairs of signals show no symmetry at all, this results in a value close to 0. These three values are multiplied together and the result is normalized to 1000. While the re-identification was performed, the resulted score value is the common (decadic) logarithm of this result. The maximum obtainable score value is 3 (=log 1000). Score value higher than 2.0 can be considered as a probable classification. The score value range (2.30–3.00) means highly probable species identification. The score value range (2.00–2.29) means secure genus identification and probable species identification.

The single spectrum gel view of each archaea strain was picked from the spectra to create a database and analyzed using the ClinPro Tools version 3.0 (Bruker Daltonics).

### Biomarker identification

The distinct mass information which could be used as identification markers was submitted to a web-based TagIdent software tool (http://web.expasy.org/tagident/) using 1% mass error for the taxonomic selections. The restrictions on protein isoelectric point were not used. This software identifies proteins based on the experimental masses acquired by MALDI mass spectrometry using the information available at the UniProtKB/Swiss-Prot and UniProtKB/TrEMBL protein sequence databases. The predicted protein mass which was closest the observed mass was chosen and presented. The molecular weight of proteins downloaded from NCBI protein database was calculated by the Compute pI/Mw tool (http://web.expasy.org/compute_pi/).

### Phylogenetic analysis with 16S rRNA sequences

The 16S rRNA sequences of the archaea type strains were downloaded from NCBI nucleotide database. Amplification and sequencing of the 16S rRNA of the other methanoarchaea used in this study was conducted as described by Wu *et al.*^27^. Procedures to amplify the other haloarchaea 16S rRNA were as previously described^42^. Multiple sequence alignments were analyzed using ClustalW of MEGA5 (http://www.megasoftware.net/)^43^, and phylogenetic trees were created using the neighbor-joining method of MEGA5.

## Additional Information

**How to cite this article**: Shih, C.-J. *et al.* Rapid identification of haloarchaea and methanoarchaea using the matrix assisted laser desorption/ionization time-of-flight mass spectrometry. *Sci. Rep.* **5**, 16326; doi: 10.1038/srep16326 (2015).

## Supplementary Material

Supplementary Information

Supplementary Dataset 1

## Figures and Tables

**Table 1:** List of haloarchaea and methanoarchaea used in this study as well as the growth conditions, accession numbers of 16S rRNA genes, and re-identified scores.

Organism	Medium	NaCl (%)	Methanogenesis substrate	Accession number	Re-identified score
Haloarchaea					
*Haloarcula argentinensis* ATCC 700875^T^	NHA	24	–	NR_116080	2.673 ± 0.019
*Haloarcula hispanica* ATCC 33960^T^	NHA	24	–	NR_113419	2.601 ± 0.033
*Haloarcula japonica* ATCC 49778^T^	NHA	24	–	NR_116082	2.314 ± 0.067
*Haloarcula marismortui* ATCC 43049^T^	NHA	24	–	NR_121590, AY596298^a^, NR_074201	2.504 ± 0.025
*Haloarcula quadrata* ATCC 700850^T^	NHA	24	–	EF645689, EF645694	2.479 ± 0.016
*Haloarcula vallismortis* ATCC 29715^T^	NHA	24	–	NR_113424	2.569 ± 0.018
*Natrinema pellirubrum* JCM 10476^T^	NHA	24	–	NR_113528	2.141 ± 0.054
*Haloterrigena turkmenica* DSM 5511^T^	NHA	24	–	NR_074238	2.272 ± 0.016
*Haloterrigena thermotolerans* DSM 11552^T^	NHA	24	–	NR_113514	2.715 ± 0.030
*Haloterrigena saccharevitans* JCM 12889^T^	NHA	24	–	NR_113512	2.709 ± 0.014
*Haloterrigena hispanica* DSM 18328^T^	NHA	24	–	NR_113508	2.692 ± 0.036
*Haloarcula sp*. HLR1	NHA	24	–	DQ089681	2.414 ± 0.004
*Haloarcula sp*. HLR2	NHA	24	–	DQ089682	2.403 ± 0.031
*Haloarcula sp*. HLR3	NHA	24	–	KP326318	2.196 ± 0.037
*Haloarcula sp*. HLR4	NHA	24	–	DQ089683	2.378 ± 0.017
*Haloarcula sp*. HLR5	NHA	24	–	DQ089684, DQ089685	2.263 ± 0.052
*Haloarcula sp*. HLR6	NHA	24	–	DQ089686	2.218 ± 0.128
*Natrinema sp*. HLW1	NHA	24	–	KP326314	2.528 ± 0.049
*Natrinema sp*. HLW2	NHA	24	–	KP326315	2.564 ± 0.042
*Natrinema sp*. HLW3	NHA	24	–	KP326316	2.405 ± 0.020
*Natrinema sp*. HLW4	NHA	24	–	KP326317	2.550 ± 0.029
*Haloterrigena sp*. H1	NHA	24	–	AF478471	2.402 ± 0.077
*Haloterrigena sp*. H4	NHA	24	–	AY546002	2.380 ± 0.103
*Haloterrigena sp*. H5	NHA	24	–	KP702942	2.755 ± 0.025
*Haloterrigena sp*. H6	NHA	24	–	AY546107	2.725 ± 0.048
*Haloterrigena sp*. H7	NHA	24	–	KP702943	2.535 ± 0.060
*Haloterrigena sp*. H8	NHA	24	–	KP702944	2.582 ± 0.055
*Haloterrigena sp*. H9	NHA	24	–	KP702945	2.662 ± 0.060
*Haloterrigena sp*. H10	NHA	24	–	KP702946	2.470 ± 0.085
*Haloterrigena sp*. H11	NHA	24	–	KP702947	2.413 ± 0.037
*Haloterrigena sp*. H12	NHA	24	–	KP702948	2.558 ± 0.015
*Haloterrigena sp*. H13	NHA	24	–	AY055733	2.390 ± 0.009
Halophilic methanoarchaea					
*Methanohalophilus portucalensis* FDF1^T^	H-P	12	40 mM trimethylamine	NR_042826	2.676 ± 0.015
*Methanohalophilus portucalensis* FDF2	H-P	12	40 mM trimethylamine	KT285318	2.655 ± 0.010
*Methanohalophilus* sp. RET-1	H-P	12	40 mM trimethylamine	KT285314	2.552 ± 0.028
*Methanohalophilus* sp. SF-1	H-P	12	40 mM trimethylamine	KT285312	2.669 ± 0.035
*Methanohalophilus* sp. SF-2	H-P	12	40 mM trimethylamine	KT285313	2.597 ± 0.027
*Methanohalophilus portucalensis* Z-7302	H-P	12	40 mM trimethylamine	KT285311	2.370 ± 0.019
*Methanohalophilus portucalensis* Z-7401	H-P	12	40 mM trimethylamine	KT285317	2.561 ± 0.037
*Methanohalophilus* sp. Z-7404	H-P	12	40 mM trimethylamine	KT285315	2.573 ± 0.016
*Methanohalophilus portucalensis* Z-7301	H-P	12	40 mM trimethylamine	KT285316	2.506 ± 0.005
*Methanohalophilus mahii* DSM 5219^T^	H-P	12	40 mM trimethylamine	M59133	2.350 ± 0.047
*Methanococcoides methylutens* Cas-1	H-P	4	40 mM trimethylamine	KT285309	2.667 ± 0.019
*Methanolobus taylorii* GS-16 ^T^	H-P	4	40 mM trimethylamine	KT285308	2.640 ± 0.026
*Methanohalobium evestigatum* SD-1	H-P	12	40 mM trimethylamine	KT285310	2.297 ± 0.018
Halotolerant and non-halophilic methanoarchaea					
*Methanolobus vulcani* DSM 3029^T^	MB/W	0.5	50 mM methanol	NR_044768	2.574 ± 0.047
*Methanolobus chelungpuianus* St545Mb^T^	MB/W	0.5	50 mM methanol	EU293796	2.312 ± 0.003
*Methanofollis formosanus* ML15^T^	MB/W	0.5	50 mM formate + 20 mM acetate	NR_042767	2.594 ± 0.047
*Methanofollis aquaemaris* N2F9704^T^	MB/W	0.5	50 mM formate + 20 mM acetate	NR_041801	2.671 ± 0.011
*Methanocorpusculum sinense* DSM 4274^T^	MB/W	0.5	50 mM formate + 20 mM acetate	NR_117148	2.193 ± 0.089
*Methanocorpusculum parvum* DSM 3823^T^	MB/W	0.5	50 mM formate + 20 mM acetate	NR_044728	2.501 ± 0.030
*Methanocorpusculum parvum* IF9601b	MB/W	0.5	50 mM formate + 20 mM acetate	AY057068	2.444 ± 0.023
*Methanocorpusculum labreanum* DSM 4855^T^	MB/W	0.5	50 mM formate + 20 mM acetate	AY260436	2.441 ± 0.019
*Methanocorpusculum bavaricum* DSM 4179^T^	MB/W	0.5	50 mM formate + 20 mM acetate	AY196676	2.475 ± 0.044
*Methanoculleus chikugoensis* Afa-1	MB/W	0.5	50 mM formate + 20 mM acetate	KP702949	2.731 ± 0.013
*Methanobacterium palustre* FG694aF	MB/W	0.5	50 mM formate + 20 mM acetate	EU293795	2.302 ± 0.020
*Methanosarcina mazei* O1M9701	MB/W	0.5	50 mM methanol	AF411469	2.649 ± 0.008
*Methanosarcina mazei* O1M9704b	MB/W	0.5	50 mM methanol	AF411467	2.471 ± 0.021
*Methanosarcina mazei* O1M9702	MB/W	0.5	50 mM methanol	AF411468	2.783 ± 0.006
*Methanosarcina mazei* FA9604c	MB/W	0.5	40 mM TMA + 20 mM acetate	AF262036	2.615 ± 0.063
*Methanococcus voltaei* P2F9701a	MB/W	0.5	50 mM formate + 20 mM acetate	AF306670	2.266 ± 0.041
*Methanocalculus taiwanensis* P2F9705	MB/W	0.5	50 mM formate + 20 mM acetate	AF411470	2.609 ± 0.036
*Methanocalculus taiwanensis* P2F9704a^T^	MB/W	0.5	50 mM formate + 20 mM acetate	AF172443	2.600 ± 0.038
*Methanocalculus pumilus* MHT-1^T^	MB/W	0.5	50 mM formate + 20 mM acetate	NR_028148	2.751 ± 0.035
*Methanocalculus halotolerans* SEBR 4845^T^	MB/W	5.0	50 mM formate + 20 mM acetate	NR_024870	2.713 ± 0.024
*Methanocalculus* sp. O1F9702c	MB/W	0.5	50 mM formate + 20 mM acetate	AY026256	2.446 ± 0.038
*Methanocalculus chunghsingensis* K1F9705b^T^	MB/W	0.5	50 mM formate + 20 mM acetate	NR_041828	2.490 ± 0.043
*Methanocalculus chunghsingensis* O1F9704a	MB/W	0.5	50 mM formate + 20 mM acetate	AY234332	2.540 ± 0.065
*Methanocalculus chunghsingensis* K1F9705c	MB/W	0.5	50 mM formate + 20 mM acetate	AF321115	2.520 ± 0.020

^a^The sequence of rrnaB was revealed from whole genome sequence.

**Table 2:** Tentative assignment of putative biomarkers from MALDI-TOF MS signals of haloarchaea type strains and methanoarchaea with genome data.

Species	UniProt Accession ID	Predicted Mass (Da)	Observed Mass (Da)/Intensity (%)	Error (%)	Protein Description	Peptide sequences
*Haloarcula argentinensis* ATCC 700875^T^	M0KI28	6143.86	6148.52/100	0.07	Uncharacterized protein	MVHCPDCETSLETADDIDFVEVDAVTGFIKASKRFYTANCAACGVTIGSGVA GAKSNGGAA
*Haloarcula hispanica* ATCC 33960^T^	G0HR47	6151.92	6148.08/100	0.06	Uncharacterized protein	MVHCPDCQTSLETADDIEFVEVDAKTGLIKASKRFYTANCAACGVTIGSGVA GAKSNGGAA
*Haloarcula japonica* ATCC 49778^T^	EMA34946^a^	6144.84	6148.70/84	0.06	hypothetical protein C444_00145	MVHCPDCQTSLETADDIDFVEVDATTGFIKASKRFYTANCAACGVTIGSGVA GAKSNGGAA
*Haloarcula marismortui* ATCC 43049^T^	Q5V7R0	6152.00	6147.87/100	0.07	Uncharacterized protein	MPEFRVRKPDGWTTVSFPDEVATISVVGGKVDGQLCLTFTGEREGGTSVVLD RLLPS
	YP_137385^a^	6159.86	6164.05/71	0.07	hypothetical protein rrnAC2944	MVHCPDCETSLETADDIEFVEVDATTGFIKASKRFYTANCAACGVTIGSGVAG AKSNGGAA
*Haloarcula vallismortis* ATCC 29715^T^	M0JP93	6148.31	6148.62/100	0.01	Uncharacterized protein	MVARLYSATLFALYQLTLLLGIMLLPVAMVTEQFGLRLPMDRAVSGLNEAYD QASA
	M0JI28	6187.91	6165.92/47	0.36	Uncharacterized protein	MVHCPDCETSLETADDIEFVEVDAVTGFIKASKRFYTANCATCGVTIGSGVAG AKSNGGAA
*Natrinema pellirubrum* JCM 10476^T^	L0JI54	6025.64	6030.57/100	0,08	Uncharacterized protein	MTPEATPVGREADRASDVVGAVDEIDGRPHLVVADIARDDAWIAMAESAAV AVEDHR
*Haloterrigena turkmenica* DSM 5511^T^	D2RXY3	6034.80	6045.45/100	0.17	Uncharacterized protein	MSTTSPVFCYVCNEEMVLDETLEHHLVYEHKPRELAKQLVAEWEAEELGEAV
	YP_ 003403049^a^	6127.76	6070.09/24	0.93	hypothetical protein Htur_1490	MTLEAESVGSVSVTDGDVVAAIDEIGGQPHLVIADIGRDDVWLSMTERDAVS LDEWR
*Haloterrigena thermotolerans* DSM 11552^T^	M0BND2	6039.66	6031.49/100	0.14	Uncharacterized protein	MTPEATPVGREADRASDVVAAVDEIDGRPHLVVADIARDDAWIAMAESAAV AVEDHR
*Methanosarcina mazei* FA9604c	Q8PYQ4^b^	10679.59	10684.8/34	0.04	50S ribosomal protein L31e	MVGKMADDMVKEQIYTIPLREVRKVPAWKRAGRAVKEVRGFLVRH MKTEAEQVKLDKTINECLWEKGCEKPPLSIRVRAVKFADGEVQAELAQ
*Methanococcus voltaei* P2F9701a	D7DR73^c^	9764.07	9751.67/43	0.13	50S ribosomal protein L12	MEYIYAALLLNSADKEITEDAVKAVLTAAGIEADDARVKALVAALEGVDIAE AIAKAAAAPVAVAAAAPAAEAPAEEKKEEKKEDTGAAAAAGLGALFG
	D7DV66^c^	9740.40	9751.67/43	0.12	50S ribosomal protein L31e	MENERIYTIPLRDVTNKVPTTKRAPRAIKKIREYLQKHMKSDNVKLDNSIN EKVWERSLNKIPARVRVKAVKQDDVVIATLVE
*Methanohalophilus mahii* DSM 5219^T^	D5E990	11436.11	11433.53/20	0.02	UPF0235 protein Mmah_0207	MPIRDAIHTKGNGCIIDFEINPGSSKLVVPSGYNIWRKRVEGKLTESAQKGK ANDQLIQRLSHIFQINSSSITIVAGAKTTKKSVHLENVYPKTAEDVLEQYL

^a^Protein accession number from NCBI protein database.

^b^The protein was described from *Methanosarcina mazei* Gö1^T^.

^c^The protein was described from *Methanococcus voltaei* A3^T^.
