# Supplementary material for: Rapid identification of haloarchaea and methanoarchaea using the matrix assisted laser desorption/ionization time-of-flight mass spectrometry
Source: Sci Rep. 2015 Nov 6;5:16326. doi: 10.1038/srep16326 (PMC4635381; doi:10.1038/srep16326)
Supplement: Supplementary Information [file srep16326-s1.pdf]

## Supplementary information

# Rapid identification of haloarchaea and methanoarchaea using the matrix assisted laser desorption/ionization time-of-flight mass spectrometry

Chao-Jen Shih[1], Sheng-Chung Chen[2], Chieh-Yin Weng[2], Mei-Chin Lai[2,3,4]* & Yu-Liang Yang[1,4]*

[1]Agricultural Biotechnology Research Center, Academia Sinica, Taipei, Taiwan

[2]Department of Life Sciences, National Chung Hsing University, Taichung, Taiwan;

[3]Agricultural Biotechnology Center, National Chung Hsing University, Taichung, Taiwan

[4]Ph.D. Program in Microbial Genomics, National Chung Hsing University and Academia Sinica, Taiwan

*Correspondence

Yu-Liang Yang: ylyang@gate.sinica.edu.tw

Mei-Chin Lai: mclai@dragon.nchu.edu.tw

**Table of Contents**


| | | |
|---|---|---|
| **Fig. S1** | Phylogenetic tree of 37 haloarchaea 16S rRNA sequences | 3 |
| **Fig. S2** | Phylogenetic tree of 24 halotorelent and non-halophilic methanoarchaea 16S rRNA sequences | 4 |
| **Fig. S3** | Phylogenetic tree of 13 halophilic methanogens 16S rRNA sequences | 5 |
| **Fig. S4** | MALDI-TOF mass spectra of *Methanohalophilus portucalensis* FDF1$^T$ were selected to establish the database | 6 |
| **Fig. S5** | MALDI-TOF mass spectra of subculturing *Methanohalophilus portucalensis* FDF1$^T$ were used to against the database | 7 |
| **Table S1** | Similarity matrix of 13 halophilic methanoarchaea 16S rRNA sequences | 8 |

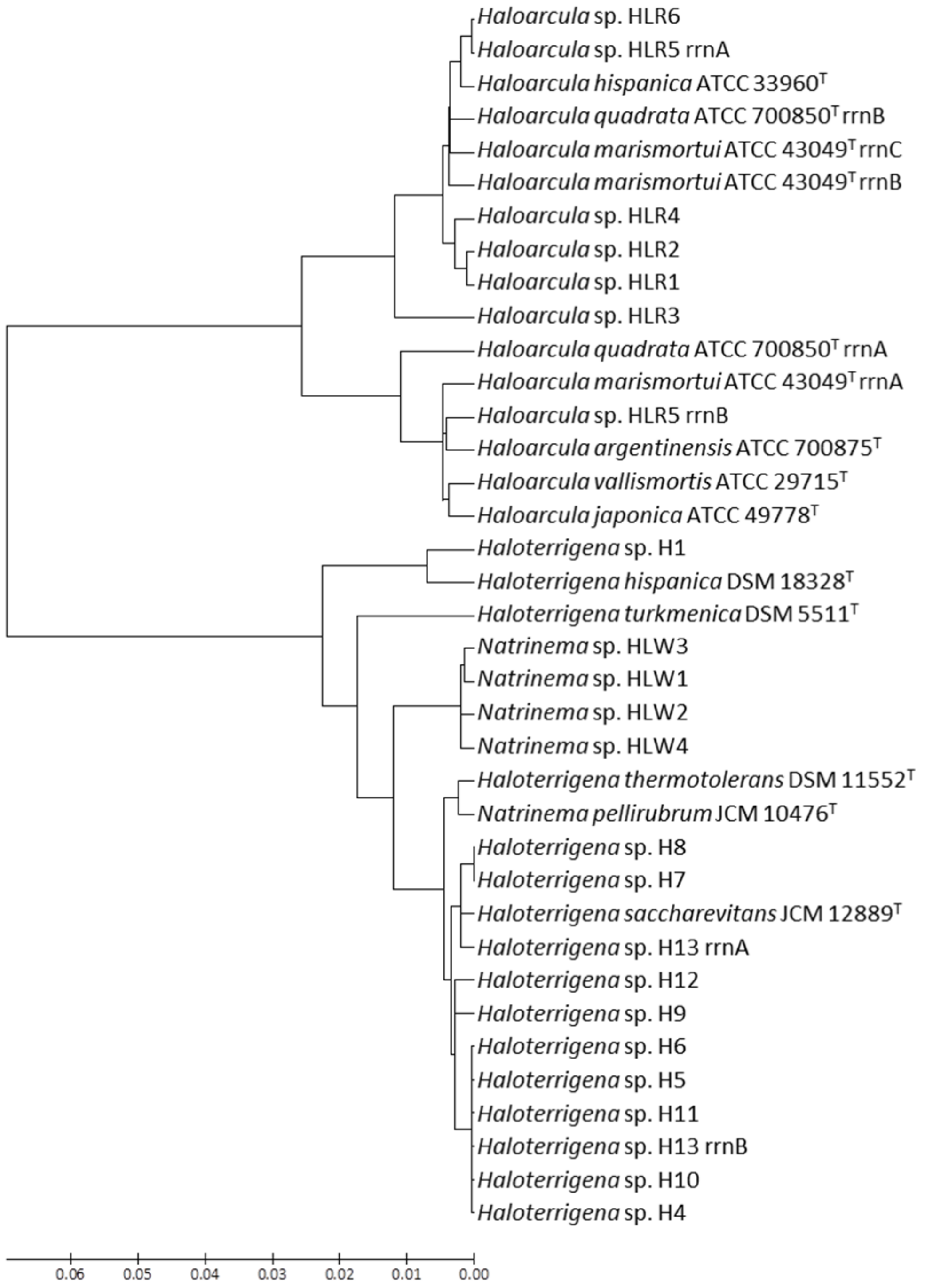


**Fig. S1. Phylogenetic tree of total 37 haloarchaea 16S rRNA sequences included the different copies from one strains.** The evolutionary history was inferred using the Neighbor-Joining method. The optimal tree with the sum of branch length = 0.31769326 is shown. The tree is drawn to scale, with branch lengths in the same units as those of the evolutionary distances used to infer the phylogenetic tree. The evolutionary distances were computed using the Maximum Composite Likelihood method and are in the units of the number of base substitutions per site. The analysis involved 37 nucleotide sequences. All positions containing gaps and missing data were eliminated. There were a total of 1312 positions in the final dataset. Evolutionary analyses were conducted in MEGA5.

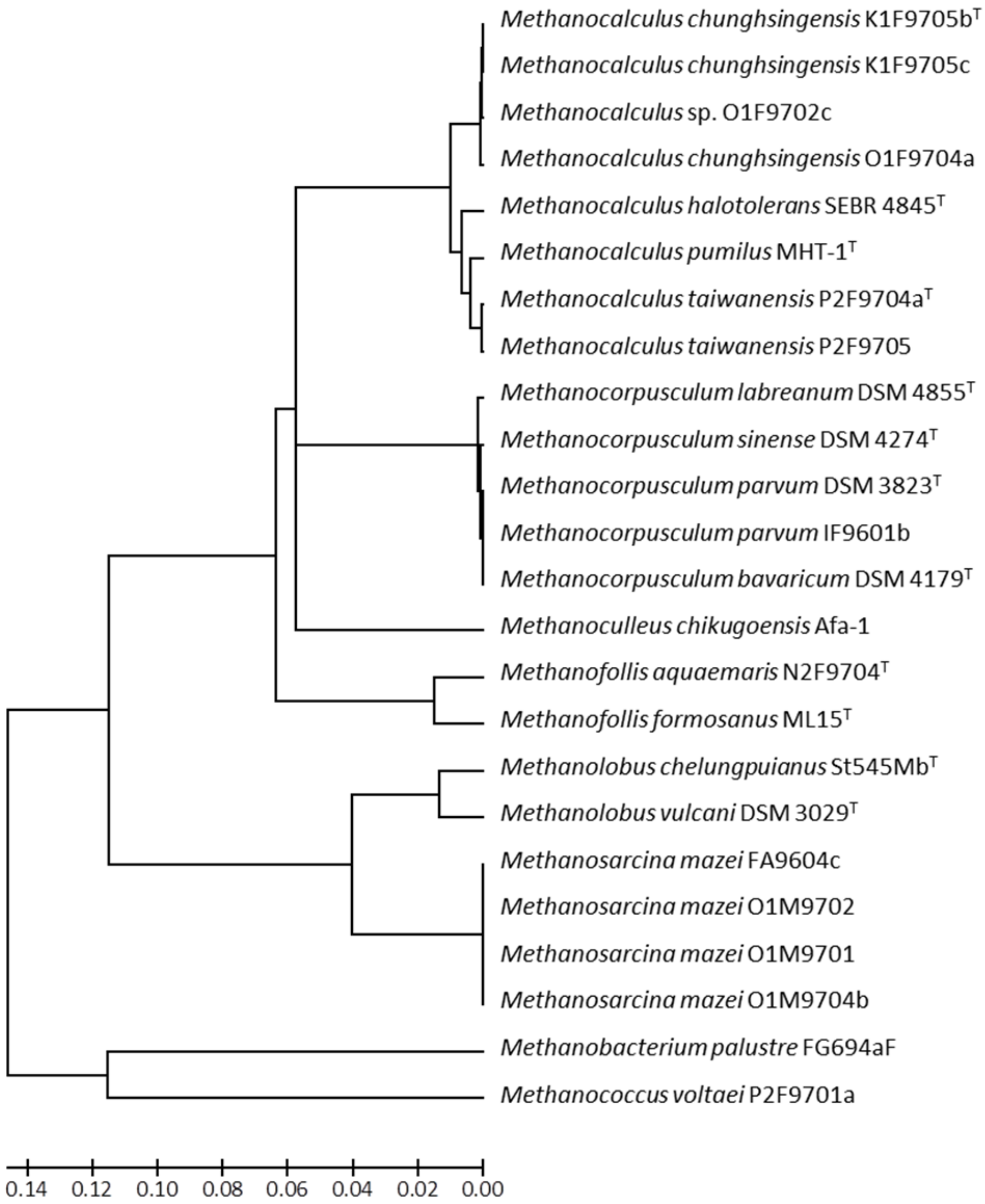


**Fig. S2. Phylogenetic tree of 24 halotorelent and non-halophilic methanogens 16S rRNA sequences.** The evolutionary history was inferred using the Neighbor-Joining method. The optimal tree with the sum of branch length = 0.77133833 is shown. The analysis involved 24 nucleotide sequences. All positions containing gaps and missing data were eliminated. There were a total of 1097 positions in the final dataset. Evolutionary analyses were conducted in MEGA5.

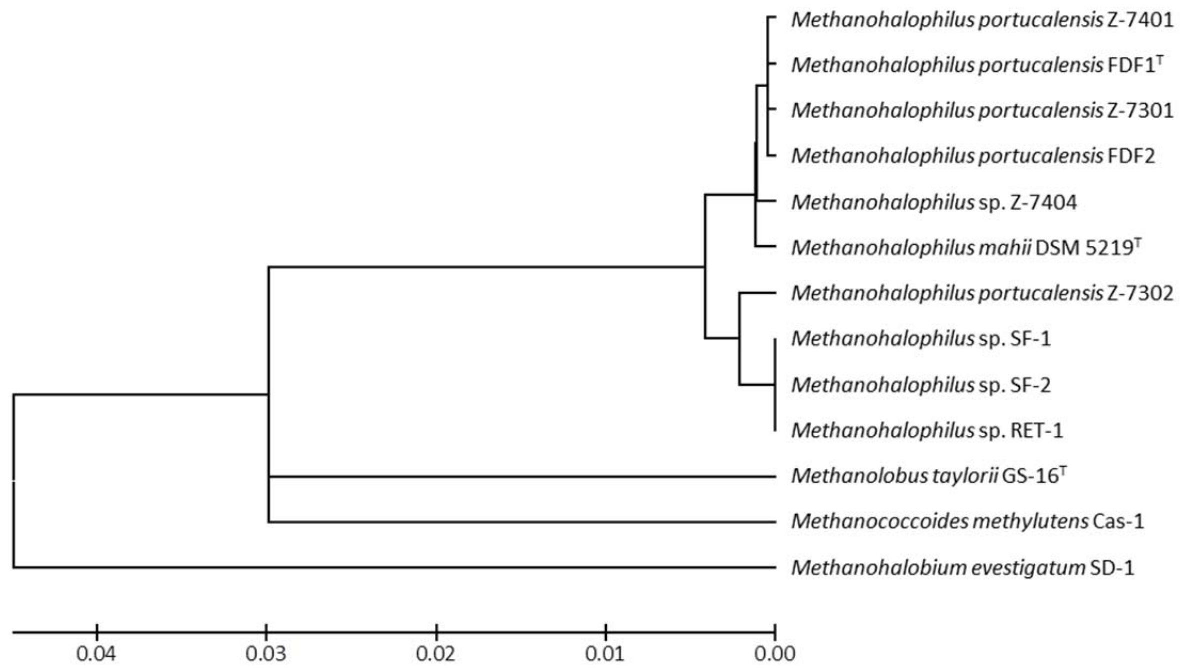


**Fig. S3. Phylogenetic tree of 13 halophilic methanogens 16S rRNA sequences.** The evolutionary history was inferred using the Neighbor-Joining method. The optimal tree with the sum of branch length = 0.15665621 is shown. The analysis involved 13 nucleotide sequences. All positions containing gaps and missing data were eliminated. There were a total of 1205 positions in the final dataset. Evolutionary analyses were conducted in MEGA5.

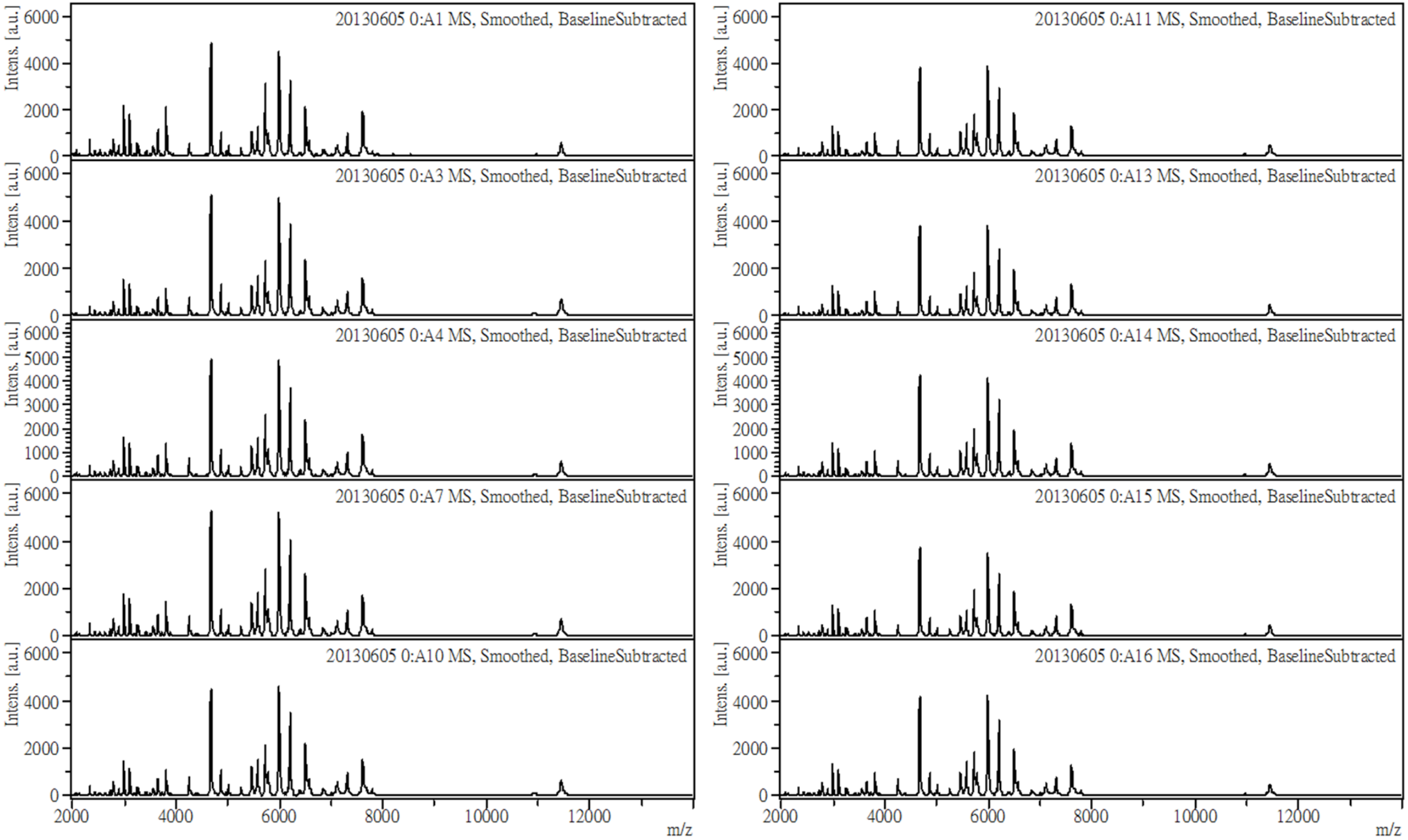


**Fig. S4. MALDI-TOF mass spectra of *Methanohalophilus portucalensis* FDF1$^{T}$ were selected to establish the database.**

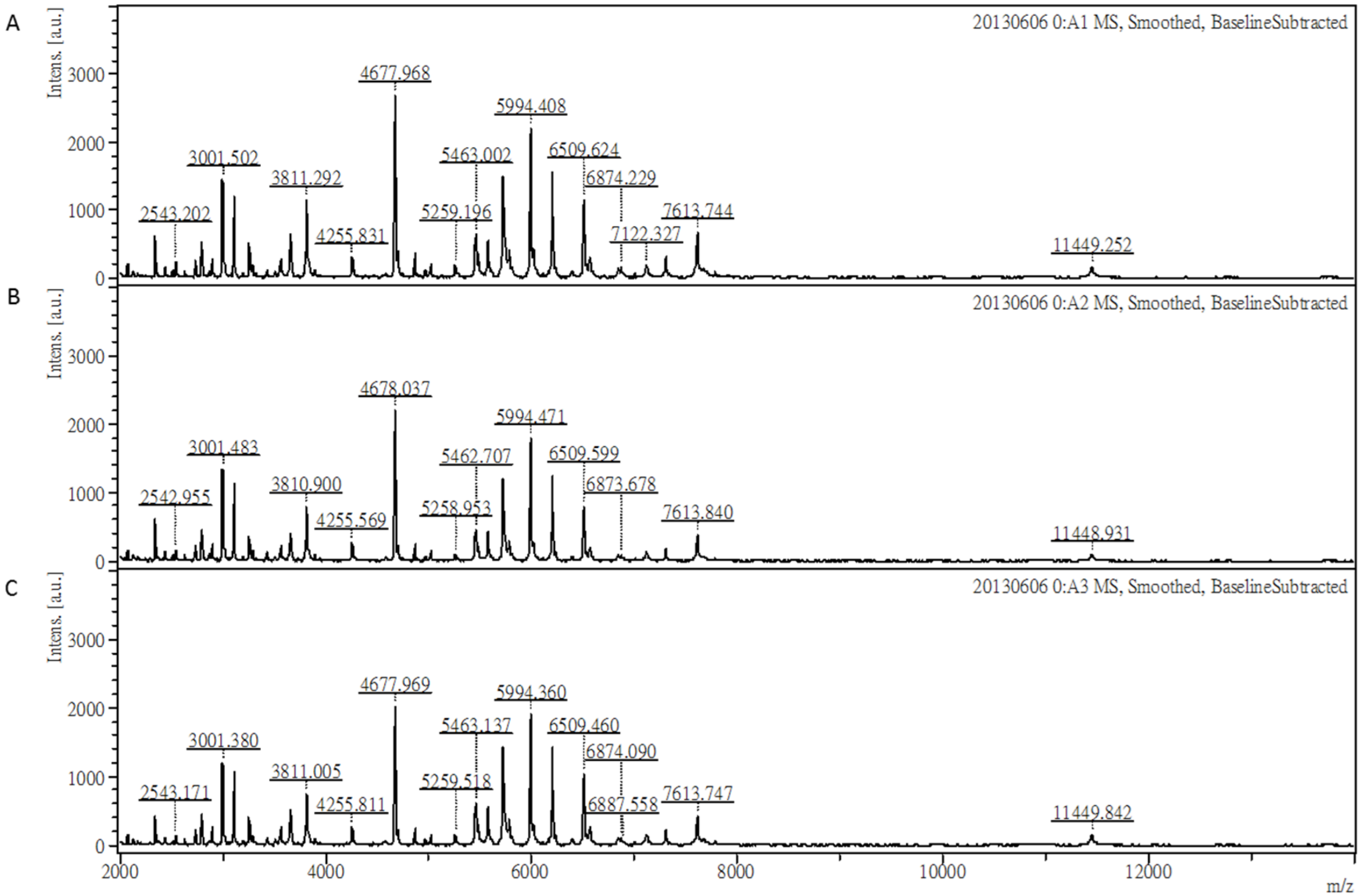


**Fig. S5. MALDI-TOF mass spectra of subculturing *Methanohalophilus portucalensis* FDF1[T] were used to against the database.** The identified score values of the triplicated spectra were A) 2.674, B) 2.696 and C) 2.659.

**Table S1. Similarity matrix of 13 halophilic methanoarchaea 16S rRNA sequences (%)**

| | | 1 | 2 | 3 | 4 | 5 | 6 | 7 | 8 | 9 | 10 | 11 | 12 | 13 |
|---|---|---|---|---|---|---|---|---|---|---|---|---|---|---|
| 1 | *Methanohalophilus portucalensis* FDF1$^T$ | | 0.25 | 0.08 | 0.08 | 0.25 | 0.25 | 0.85 | 0.76 | 0.76 | 0.76 | 5.89 | 5.91 | 8.95 |
| 2 | *Methanohalophilus portucalensis* Z-7401 | 99.75 | | 0.17 | 0.17 | 0.34 | 0.34 | 0.93 | 0.85 | 0.85 | 0.85 | 5.98 | 6.00 | 9.04 |
| 3 | *Methanohalophilus portucalensis* Z-7301 | 99.92 | 99.83 | | 0.00 | 0.17 | 0.17 | 0.76 | 0.68 | 0.68 | 0.68 | 5.80 | 5.81 | 8.85 |
| 4 | *Methanohalophilus portucalensis* FDF2 | 99.92 | 99.83 | 100.00 | | 0.17 | 0.17 | 0.76 | 0.68 | 0.68 | 0.68 | 5.80 | 5.81 | 8.85 |
| 5 | *Methanohalophilus mahii* DSM 5219$^T$ | 99.75 | 99.66 | 99.83 | 99.83 | | 0.25 | 0.76 | 0.68 | 0.68 | 0.68 | 5.71 | 5.82 | 8.84 |
| 6 | *Methanohalophilus* sp. Z-7404 | 99.75 | 99.66 | 99.83 | 99.83 | 99.75 | | 0.85 | 0.76 | 0.76 | 0.76 | 5.62 | 5.82 | 8.94 |
| 7 | *Methanohalophilus porucalensis* Z-7302 | 99.15 | 99.07 | 99.24 | 99.24 | 99.24 | 99.15 | | 0.34 | 0.34 | 0.34 | 6.35 | 6.27 | 9.32 |
| 8 | *Methanohalophilus* sp. SF-1 | 99.24 | 99.15 | 99.32 | 99.32 | 99.32 | 99.24 | 99.66 | | 0.00 | 0.00 | 6.26 | 6.27 | 9.23 |
| 9 | *Methanohalophilus* sp. SF-2 | 99.24 | 99.15 | 99.32 | 99.32 | 99.32 | 99.24 | 99.66 | 100.00 | | 0.00 | 6.26 | 6.27 | 9.23 |
| 10 | *Methanohalophilus* sp. RET-1 | 99.24 | 99.15 | 99.32 | 99.32 | 99.32 | 99.24 | 99.66 | 100.00 | 100.00 | | 6.26 | 6.27 | 9.23 |
| 11 | *Methanococcoides methylutens* MM1 | 94.11 | 94.02 | 94.20 | 94.20 | 94.29 | 94.38 | 93.65 | 93.74 | 93.74 | 93.74 | | 6.29 | 7.69 |
| 12 | *Methanohalophilus taylorii* GS-16$^T$ | 94.09 | 94.00 | 94.19 | 94.19 | 94.18 | 94.18 | 93.73 | 93.73 | 93.73 | 93.73 | 93.71 | | 8.16 |
| 13 | *Methanohalobium evestigatum* SD-1 | 91.05 | 90.96 | 91.15 | 91.15 | 91.16 | 91.06 | 90.68 | 90.77 | 90.77 | 90.77 | 92.31 | 91.84 | |
